# Risk factors of symptomatic anastomotic leakage and its impacts on a long-term survival after laparoscopic low anterior resection for rectal cancer: a retrospective single-center study

**DOI:** 10.1186/s12957-021-02303-5

**Published:** 2021-06-25

**Authors:** Xinyu Qi, Maoxing Liu, Kai Xu, Pin Gao, Fei Tan, Zhendan Yao, Nan Zhang, Hong Yang, Chenghai Zhang, Jiadi Xing, Ming Cui, Xiangqian Su

**Affiliations:** Key Laboratory of Carcinogenesis and Translational Research (Ministry of Education), Department of Gastrointestinal Surgery IV, Peking University Cancer Hospital and Institute, Beijing, 100142 People’s Republic of China

**Keywords:** Symptomatic anastomotic leakage, Risk factors, Long-term survival, Laparoscopic low anterior resection, Rectal cancer

## Abstract

**Background:**

Postoperative symptomatic anastomotic leakage (AL) is a serious complication after low anterior resection (LAR) for rectal cancer. AL can potentially affect short-term patient outcomes and long-term prognosis. This study aimed to explore the risk factors and long-term survival of symptomatic AL after laparoscopic LAR for rectal cancer.

**Methods:**

From May 2009 to May 2015, 298 consecutive patients who underwent laparoscopic LAR for rectal cancer with or without a defunctioning stoma were included in this study. Univariate and multivariate logistic regression analyses were used to explore independent risk factors for symptomatic AL. Survival analysis was performed using Kaplan–Meier curves, and log-rank tests were used for group comparisons.

**Results:**

Among the 298 patients enrolled in this study, symptomatic AL occurred in eight (2.7%) patients. The univariate analysis showed that age of ≤65 years (*P* = 0.048), neoadjuvant therapy (*P* = 0.095), distance from the anal verge (*P* = 0.078), duration of operation (*P* = 0.001), and pathological tumor (T) category (*P* = 0.004) were associated with symptomatic AL. The multivariate analysis demonstrated that prolonged duration of operation (*P =* 0.010) was an independent risk factor for symptomatic AL after laparoscopic LAR for rectal cancer. No statistically significant differences were observed in the 3-year (*P* = 0.785) and 5-year (*P* = 0.979) overall survival rates.

**Conclusions:**

A prolonged duration of operation increased the risk of symptomatic AL after laparoscopic LAR for rectal cancer. An impact of symptomatic AL on a long-term survival was not observed in this study; however, further studies are required.

**Trial registration:**

This study was registered in the Chinese Clinical Trial Registry (ChiCTR2000033413) on May 31, 2020.

## Background

Postoperative anastomotic leakage (AL) after low anterior resection (LAR) for rectal cancer is a common and devastating complication, and the reported incidence of AL varies from 2.2 to 18.6% [1–8]. It significantly increases mortality rates and health care costs in short time [3, 9] and also has higher local recurrence during long-term follow-up [10]. In the past 10 years, different risk factors for AL after open LAR have been reported, but few studies have exclusively focused on the risk factors of AL after laparoscopic LAR [4, 6]. Indeed, laparoscopic LAR, instead of laparotomy, has become the standard operation for rectal cancer. Kang [3] and Zheng [7] reported that laparoscopy might decrease the risk of AL because less tissue trauma occurs and a better pelvis surgical field exposure can be obtained using laparoscopic technology. However, rectal transection and the double-stapling technique anastomosis using linear and circular staples are relatively difficult laparoscopic maneuvers compared with laparotomy. Moreover, the postoperative course after laparoscopic LAR is different compared with that after laparotomy [11]. Therefore, the risk factors of AL after laparoscopic LAR may differ from those after laparotomy. Furthermore, by reviewing articles published in the past 5 years, we found out that no agreement has been achieved on whether AL has an impact on oncological outcomes [10, 12, 13]. Therefore, the present study has two clinical objectives: (1) the primary objective was to identify the risk factors of symptomatic AL after laparoscopic LAR based on patient, surgery, and tumor-related variables, and (2) the secondary objective was to assess the impact of symptomatic AL on 3- and 5-year overall survival rates.

## Methods

### Patients

From May 2009 to May 2015, 312 consecutive patients with primary rectal cancer underwent laparoscopic LAR at the Department of Gastrointestinal Surgery IV, Peking University Cancer Hospital and Institute. Four patients who chose laparotomy and six patients who chose laparoscopy and then converted to laparotomy were excluded. Two patients who underwent emergency surgery due to intestinal perforation and two patients who had missing data were also excluded. Thus, 298 patients with primary rectal cancer were enrolled. Preoperative colonoscopy and pathological biopsy confirmed rectal cancer in all patients. High-resolution magnetic resonance imaging, and not endoscopic ultrasonography, is a routine examination performed on patients with low rectal cancer in our unit. All patients and their families provided written informed consent before surgery. On the basis of the total mesorectal excision principle, all patients were eligible for curative R0 resection using laparoscopic LAR. Neoadjuvant therapy was necessary for patients diagnosed with low rectal cancer who were evaluated as T3-4/N+ in our unit. Patients on neoadjuvant therapy received 50.4–54.0 Gy of radiation and 5-FU-based chemotherapy (capecitabine) before surgery. Surgery was performed 8 weeks after the completion of chemoradiation. Postoperative chemotherapy (FOLFOX 4 plan) was recommended to all patients with positive lymph nodes as described in the final pathology report. The patients were followed-up every 3 months for the first 2 years after surgery and then every 6 months thereafter. Data from the last available follow-up visit were included in the analysis. This study was approved by the Medical Ethics Committee of our institution and was conducted in accordance with principles of the Declaration of Helsinki of the World Medical Association. This study was registered in the Chinese Clinical Trial Registry (ChiCTR2000033413) on May 31, 2020. This study report is in line with the strengthening the reporting of cohort studies in surgery criteria [14].

### Surgical procedure

According to the recommended clinical pathway, all patients underwent preoperative bowel preparation 1 day before surgery. Laxatives were directly used in patients without intestinal obstruction. Patients with incomplete intestinal obstruction were given olive oil first, followed by laxatives. Prophylactic cephalosporin antibiotics were given 0.5–2 h before surgery and were continued to be administered for 1 day after surgery. All procedures were successively conducted by three experienced colorectal surgeons from the same group in our unit. Laparoscopic LAR was performed as reported by Zhou et al [5]. End-to-end anastomosis was used for colorectal reconstruction and was performed with a circular stapler (ETHICONTM Circular Stapler CDH25A; Ethicon Endo-Surgery LLC). The integrity of the ring of tissue retained by the circular stapler after performing the anastomosis was routinely examined. Air leak test was selectively performed to assess the integrity of the anastomosis for patients in our unit, particularly patients with incomplete doughnut ring after anastomosis. A defunctioning stoma was selectively performed at the discretion of the surgeon. Generally, stoma was performed if any of the following adverse events occurred: (1) low level of anastomosis (≤3cm from the anal verge); (2) neoadjuvant therapy before surgery, particularly neoadjuvant radiotherapy; (3) positive air leak test; (4) tissue edema, particularly patients with intestinal obstruction before surgery; or (5) an incomplete doughnut ring no matter the size of the defect. The transanal tube drain was mainly used to patients with preoperative intestinal obstruction and larger tension after anastomosis judged in surgery. The pelvic drain was routinely placed next to the anastomosis, and the transanal tube drain was placed according to the status of the patient and the discretion of the surgeon. The pelvic drain was removed when the output of the drain was clear and <50 mL/24 h. The transanal tube drain was removed approximately 3 days after surgery.

### Definitions and variables

In the present study, we used the standard definition of AL to guarantee the accuracy of the diagnosis. On the basis of the International Study Group of Rectal Cancer (ISREC) recommendations of 2010, AL was defined as a defect at the anastomotic site leading to a communication between intraluminal and extraluminal compartments [15]. On the basis of the severity and clinical symptoms, AL was classified into three grades (A, B, and C). Grade A leaks are those diagnosed with slight radiological evidence that do not require treatment. Grade B leaks are those managed with nonoperative treatment. Grade C leaks are those requiring a second surgical intervention. In our unit, the diagnosis of AL mainly depends on clinical symptoms and signs, characteristics of drainage fluid, and abdominal and pelvic plain scan or enhanced computed tomography (CT). Patients who met the criterion for grade B or C were diagnosed with symptomatic AL. Patients with grade A leaks were not included. Grade A leaks are those diagnosed barely with abdominal and pelvic plain scan or enhanced CT in our unit. Endoscopy, CT, or barium enema was not routinely adopted in our unit. The follow-up period for symptomatic AL was within 30 days after surgery. The location of the tumor and distance from the anal verge were determined by CT or colonoscopy and confirmed during surgery. Rectal cancer histopathological staging was defined according to the Union for International Cancer Control-TNM classification (8th edition) [16].

**Fig. 1:**
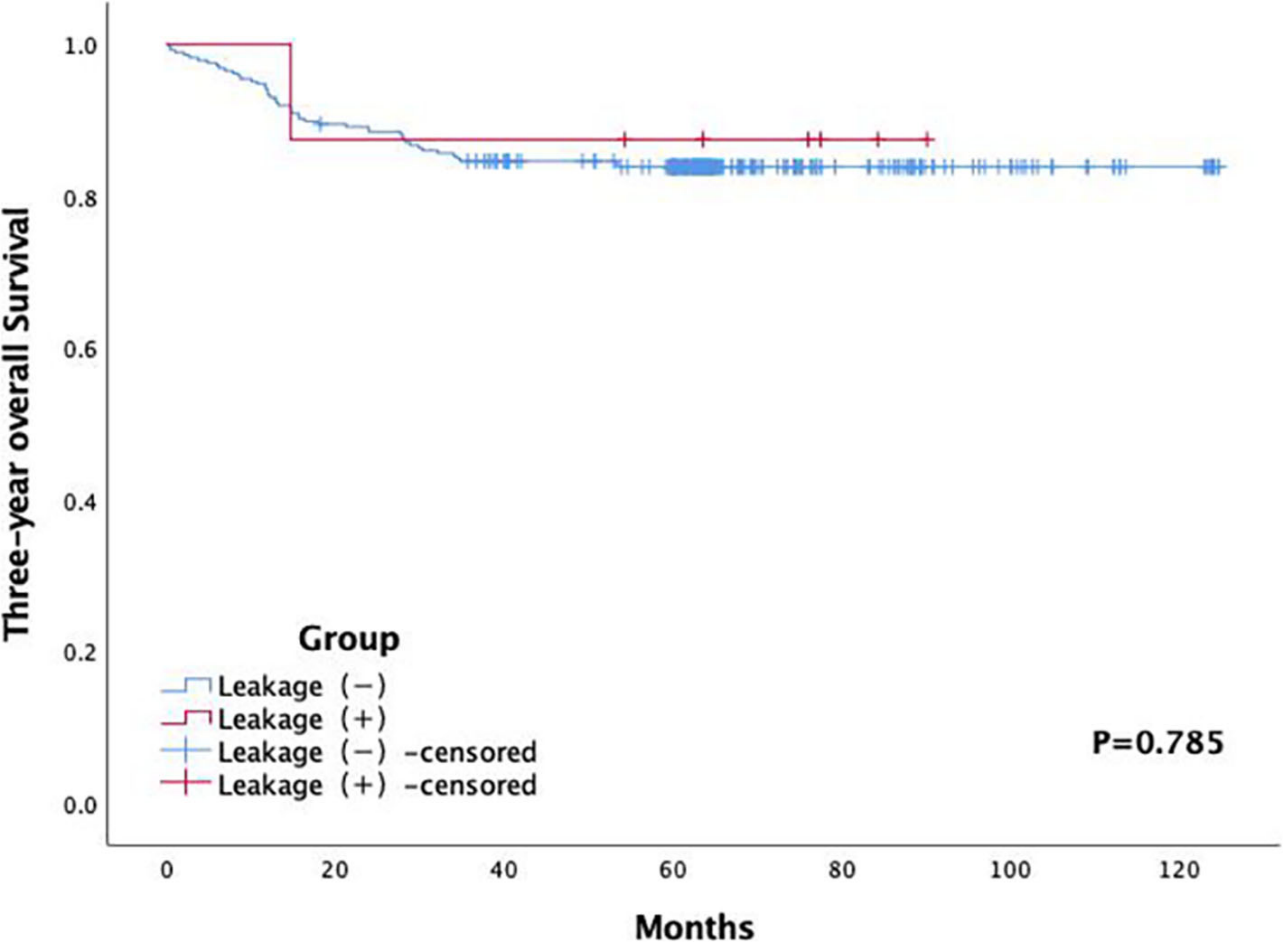
Three-year overall survival according to Kaplan–Meier analysis

**Fig. 2:**
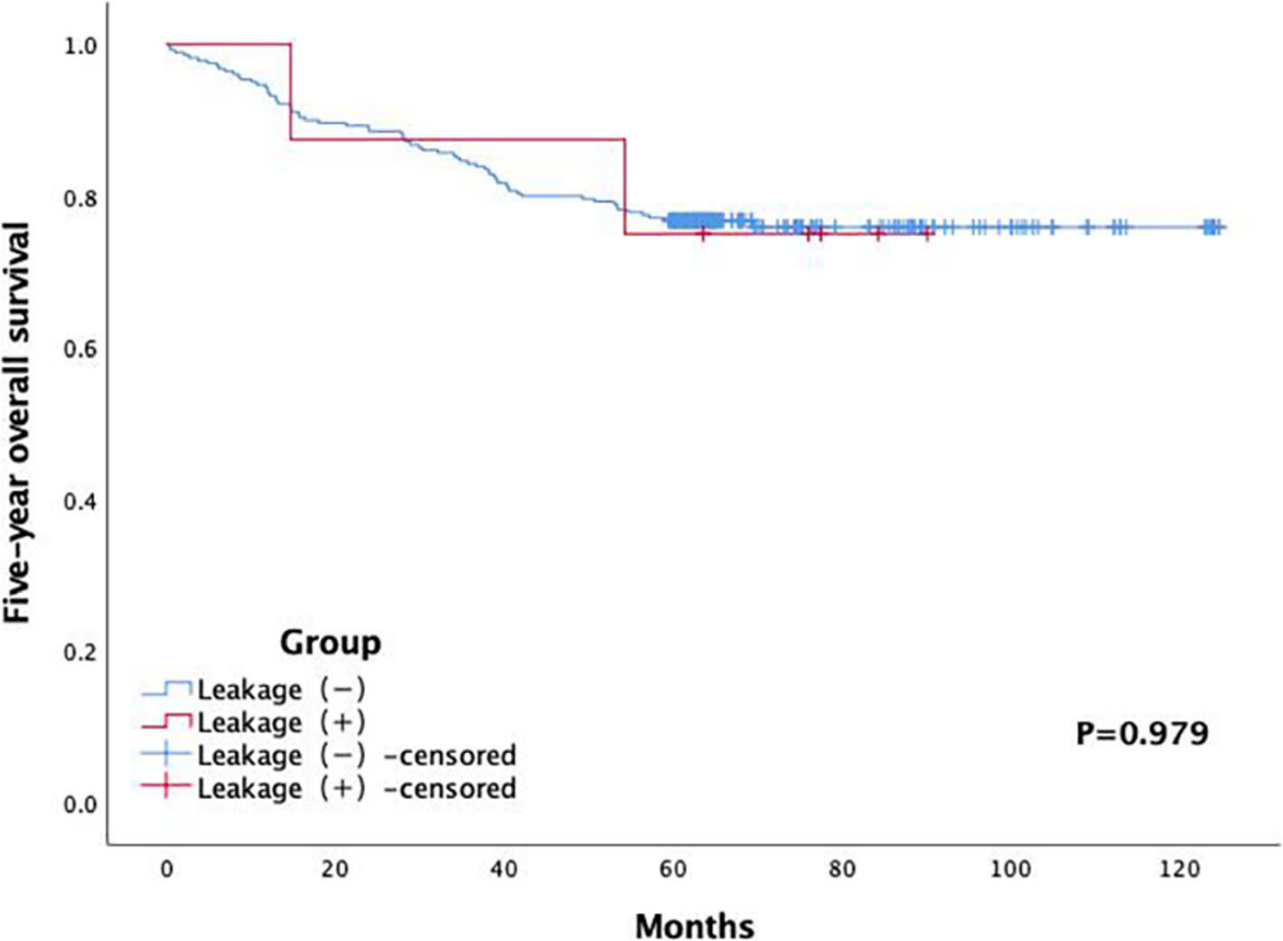
Five-year overall survival according to Kaplan–Meier analysis

Patient-related, surgery-related, and tumor-related variables potentially associated with symptomatic AL were recorded. Table 1 summarizes the patient-related variables, including the gender (male and female), age (≤65 and >65 years), American Society of Anesthesiologists (ASA) physical status classification (I–III), smoking status, alcohol consumption, previous history of abdominal surgery, preoperative intestinal obstruction, hypertension, diabetes, neoadjuvant therapy, and pulmonary insufficiency, as well as body mass index (BMI) (≤18, 18–25, or ≥25 kg/m^2^), preoperative hemoglobin level (<120 and ≥120 g/L), preoperative white blood cell count (<4000, 4000–12000, or >12000), preoperative serum albumin level (<35 and ≥35 g/L), and preoperative carcinoembryonic antigen level (<5 and ≥5 ng/mL). Table 2 summarizes the surgery-related variables, including the distance from the anal verge (<7 and ≥7 cm), duration of operation (<200 and ≥200 min), intraoperative blood loss (<100 and ≥100 mL), combined resection of other organs, perioperative blood transfusion, defunctioning stoma, and transanal tube drainage. Table 3 summarizes the tumor-related variables, including pathology type (adenocarcinoma, mucinous carcinoma, or signet-ring cell), tumor size (≤5 and >5 cm), tumor differentiation (well, moderate, poor, well-moderate, or moderate-poor), tumor type (ulcer, uplift, or infiltrating), tumor infiltration (1/4, 1/3, 1/2, 2/3, 3/4, or full circle), vascular invasion, nerve invasion, pathological tumor T category (T0–4), pathological node N category (N0–3), metastasis (M0-1), and cancerous node. In total, 34 potential risk factors were considered and analyzed. Figures 1 and 2 show the 3-year and 5-year overall survival rates and follow-up time, respectively.

**Table 1:** Univariate analysis of patient-related variables with symptomatic anastomotic leakage

Variables	AL (−) (*N*=290)	AL (+) (*N*=8)	χ^2^	*P* value
Gender			1.120	0.290
Male	163 (56.2)	6 (75)		
Female	127 (43.8)	2 (25)		
Age (years)			3.907	0.048^**†**^
≤ 65	194 (66.9)	8 (100)		
>65	96 (33.1)	0 (0)		
ASA category			1.290	0.525
I	40 (13.8)	0 (0)		
II	222 (76.6)	7 (87.5)		
III	28 (9.6)	1 (12.5)		
Smoking			0.148	0.701
No	199 (68.6)	6 (75)		
Yes	91 (31.4)	2 (25)		
Alcohol consumption			2.408	0.121
No	242 (83.4)	5 (62.5)		
Yes	48 (16.6)	3 (37.5)		
Previous history of abdominal surgery			0.175	0.676
No	237 (81.7)	7 (87.5)		
Yes	53 (18.3)	1 (12.5)		
Preoperative intestinal obstruction			0.886	0.346
No	261 (90)	8 (100)		
Yes	29 (10)	0 (0)		
Hypertension			0.796	0.372
No	213 (73.4)	7 (87.5)		
Yes	77 (26.6)	1 (12.5)		
Diabetes			0.989	0.320
No	258 (89.0)	8 (100)		
Yes	32 (11.0)	0 (0)		
BMI (kg/m^2^)			2.352	0.309
≤18	17 (5.8)	0 (0)		
18–25	140 (48.3)	6 (75)		
≥ 25	133 (45.9)	2 (25)		
Neoadjuvant therapy			2.784	0.095^**†**^
No	245 (84.5)	5 (62.5)		
Yes	45 (15.5)	3 (37.5)		
Pulmonary insufficiency			2.571	0.109
No	219 (75.5)	8 (100)		
Yes	71 (24.5)	0 (0)		
Preoperative hemoglobin(g/L)			0.898	0.343
<120	67 (23.1)	3 (37.5)		
≥120	223 (76.9)	5 (62.5)		
Preoperative white blood cell			0.591	0.442
4000–12000	270 (93.1)	8 (100)		
<4000 or >12000	20 (6.9)	0 (0)		
Preoperative serum albumin (g/L)			0.440	0.507
<35	19 (6.6)	1 (12.5)		
≥35	271 (93.4)	7 (87.5)		
Preoperative CEA (ng/ml)			0.732	0.392
<5	174 (60)	6 (75)		
≥5	116 (40)	2 (25)		

*AL* anastomotic leakage, *ASA* American Society of Anesthesiologists, *BMI* body mass index, *CEA* carcinoembryonic antigen, *χ*^*2*^ chi-square test

^†^p<0.1

**Table 2:** Univariate analysis of surgery-related variables for symptomatic anastomotic leakage

Variables	AL (−) (*N*=290)	AL (+) (*N*=8)	χ^2^	*P* value
Distance from the anal verge (cm)			3.103	0.078^**†**^
<7	67 (23.1)	4 (50)		
≥7	223 (76.9)	4 (50)		
Duration of operation(min)			11.084	0.001^**†**^
<200	222 (76.6)	2 (25)		
≥200	68 (23.4)	6 (75)		
Intraoperative blood loss(ml)			0.444	0.505
<100	225 (77.6)	7 (87.5)		
≥100	65 (22.4)	1 (12.5)		
Combined organ resection			0.560	0.454
No	271 (93.4)	8 (100)		
Yes	19 (6.6)	0 (0)		
Intraoperative blood transfusion			1.527	0.217
No	279 (96.2)	7 (87.5)		
Yes	11 (3.8)	1 (12.5)		
Defunctioning stoma			1.691	0.193
No	150 (51.7)	6 (75)		
Yes	140 (48.3)	2 (25)		
Transanal tube drainage			0.000	0.994
No	254 (87.6)	1 (12.5)		
Yes	36 (12.4)	7 (87.5)		

*AL* anastomotic leakage*, χ*^*2*^ chi-square test

^**†**^*p*<0.1

**Table 3:** Univariate analysis of tumor-related variables for symptomatic anastomotic leakage

Variables	AL (−) (*N*=290)	AL (+) (*N*=8)	χ^2^	*P* value
Pathology type of tumor			0.405	0.817
Adenocarcinoma	276 (95.2)	8 (100)		
Mucinous carcinoma	11 (3.8)	0 (0)		
Signet-ring cell	3 (1.0)	0 (0)		
Size of tumor(cm)			0.576	0.448
≤5	246 (84.8)	6 (75)		
>5	44 (15.2)	2 (25)		
Tumor differentiation			2.410	0.661
Well	19(6.6)	0 (0)		
Moderate	224 (77.2)	6 (75)		
Poor Well-moderate	9 (3.1) 7 (2.4)	0 (0) 0 (0)		
Moderate-poor	31 (10.7)	2 (25)		
Tumor type			3.526	0.172
Ulcer	182 (62.8)	7 (87.5)		
Uplift	88 (30.3)	0 (0)		
Infiltrating	20 (6.9)	1 (12.5)		
Tumor infiltrating			2.570	0.766
1/4 circle	38 (12.8)	2 (25)		
1/3 circle	79 (26.5)	1 (12.5)		
1/2 circle	68 (22.8)	2 (25)		
2/3 circle	48 (16.1)	2 (25)		
3/4 circle	27 (9.1)	0 (0)		
Full circle	30 (10.1)	1 (12.5)		
Vascular invasion			0.213	0.644
No	235 (81)	7 (87.5)		
Yes	55 (19)	1 (12.5)		
Nerve invasion			0.655	0.418
No	268 (92.4)	8 (100)		
Yes	22 (7.6)	0 (0)		
Pathological tumor(T)category			15.357	0.004^**†**^
T0	5 (1.7)	0 (0)		
T1	21(7.3)	0 (0)		
T2	55 (19)	6 (75)		
T3	163 (56.4)	1 (12.5)		
T4	45 (15.6)	1 (12.5)		
Pathological node(N)category			1.945	0.584
N0	162 (55.9)	6 (75)		
N1	76 (26.2)	2 (25)		
N2	46 (15.9)	0 (0)		
N3	6 (2.1)	0 (0)		
Metastasis			0.345	0.557
M0	278 (95.9)	8 (100)		
M1	12 (4.1)	0 (0)		
Cancerous node			1.305	0.253
No	278 (95.9)	7 (87.5)		
Yes	12 (4.1)	1 (12.5)		

*AL* anastomotic leakage, χ^2^ chi-square test

^**†**^*p*<0.1

### Symptomatic AL interventions

The interventions for symptomatic AL were as follows: (1) nonoperative treatment: dietary modification, total parenteral nutritional support, use of antibiotics, transanal tube drainage, and percutaneous drainage or (2) surgical treatment: repair, massive irrigation and drainage, and defunctioning stoma.

### Statistical analyses

Statistical analyses were performed using SPSS version 19.0 (IBM Corp., Armonk, NY, USA). Continuous variables were dichotomized according to the clinical situation, and standard values were stipulated by the state-of-the-art guidelines or using the median value of each variable as the cutoff point. All patients were divided into two groups according to the occurrence of symptomatic AL or no AL, and the groups were analyzed using the chi-squared test or Fisher’s exact test. Variables with a *P* value of <0.1 in the univariate analysis and other factors that were thought to have important clinical significance were entered into the multivariate analysis. The multivariate analysis used a logistic regression model to determine the risk factors associated with the incidence of symptomatic AL, and a *P* value of <0.05 was considered statistically significant. Survival analyses were performed using Kaplan–Meier curves, and the log-rank test was used for comparisons. A *P* value of <0.05 was considered statistically significant for each test.

## Results

### Incidence of symptomatic AL

Of the 298 consecutive patients who underwent laparoscopic LAR for rectal cancer, 169 (57.0%) were male and 129 (43%) were female. The median age was 63 (range, 23–85) years, and the median BMI was 25 (range, 16–36) kg/m^2^. Eight (2.7%) patients were diagnosed with symptomatic AL, and no grade A patients were included in this study. Of the eight patients with symptomatic AL, three (37.5%) patients were classified as grade B and treated with irrigation and drainage through the pelvic drain without a defunctioning stoma, whereas five (62.5%) were classified as grade C and treated with a defunctioning stoma in our unit. The median time of AL was 3 (range, 2–6) days after surgery, and no patients died during the perioperative period.

### Patient-related variables

Table 1 shows the results of the univariate analysis of patient-related variables for symptomatic AL. Patient characteristics, living habits, comorbidities, and nutritional status were compared and analyzed. The symptomatic AL rate was higher in males (75%) than in females (25%), although statistically significant differences were not reached (*P* = 0.290). There were statistically significant differences between the two groups in terms of age (*P* = 0.048). The proportion of patients with AL who received neoadjuvant therapy was slightly lower than that of those who did not receive neoadjuvant therapy, and the difference was statistically significant (37.5% vs. 62.5%, *P* = 0.095) in univariate analysis. Other variables, including ASA category, smoking, alcohol consumption, history of abdominal surgery, preoperative intestinal obstruction, hypertension, diabetes, and pulmonary insufficiency, as well as BMI, preoperative hemoglobin level, white blood cell, serum albumin level, and carcinoembryonic antigen level, were not significantly different in patients with symptomatic AL.

### Surgery-related variables

Table 2 shows the results of the univariate analysis of surgery-related variables in patients with symptomatic AL. There were no statistically significant differences between the two groups with regard to intraoperative blood loss, combined organ resection, intraoperative blood transfusion, defunctioning stoma, and transanal tube drainage. However, patients with a prolonged duration of surgery (≥200 min) (75% vs. 25%, *P* = 0.001) were at a higher risk of symptomatic AL than those without a prolonged duration of surgery. The proportion of patients who had tumors within 7 cm of the anal verge was equal to the proportion of patients with tumors measuring >7 cm from the anal verge (50% vs. 50%, *P =* 0.078). Moreover, of the 144 patients who had defunctioning stoma, 131 had ileostomy closures within 6–12 months after surgery, five died of tumors within 1 year after surgery, four died of other diseases, and the other four did not have closures due to health issues. No subclinical leaks were found in our unit.

### Tumor-related variables

Table 3 lists the results of the univariate analysis of the tumor-related variables for symptomatic AL. The tumor pathology type, tumor size, tumor differentiation, tumor type, tumor infiltration, vascular invasion, nerve invasion, pathological node (N) category, metastasis, and cancerous node were not identified as risk factors significantly associated with symptomatic AL. Moreover, symptomatic AL occurred in stage T2–4 tumors with statistically significant differences (*P* = 0.004). In general, all eight patients with AL had adenocarcinoma, with no differences in nerve invasion and metastasis in the symptomatic AL group compared with the no AL group.

### Multivariate analysis

Univariate analysis revealed that the age of ≤65 years (*P* = 0.048), neoadjuvant therapy (*P* = 0.095), distance from the anal verge (*P* = 0.078), duration of operation (*P* = 0.001), and pathological tumor (T) category (*P* = 0.004) were the risk factors for symptomatic AL. To adjust for confounding bias, we incorporated these and other variables that were thought to have important clinical significance in the multivariate analysis and revealed that only the duration of operation (*P* = 0.010, OR 9.058 [95% CI, 1.695–48.401]) was an independent risk factor for symptomatic AL.

### Long-term survival

The mean follow-up time was 62.42 ± 20.33 (range, 57–68) months. Three and ten patients were lost to follow-up at 3 and 5 years, respectively, without a statistical significance. There were no statistically significant differences in 3-year (*P* = 0.785) and 5-year overall survival rates (*P* = 0.979) between the two groups, as shown in Figs. 1 and 2.

## Discussion

The risk factors and a long-term survival of symptomatic AL were assessed in 298 consecutive patients undergoing laparoscopic LAR for rectal cancer. Univariate and multivariate analyses demonstrated that a prolonged duration of operation was an independent risk factor for symptomatic AL (Table 4). Moreover, no significant differences were found in the 3- and 5-year overall survival rates between the two groups.

**Table 4:** Multivariate analysis of the risk factors for symptomatic AL

Variables	Odds ratio	95 % CI	*P* value
Age (years)	–	–	0.996
Neoadjuvant therapy	1.754	0.368–8.360	0.481
Duration of operation(min)	9.058	1.695–48.401	0.010^**†**^
Distance from the anal verge (cm)	0.499	0.109–2.293	0.372
Pathological tumor(T)category	0.765	0.347–1.684	0.505

*AL* anastomotic leakage, *CI* confidence interval

^**†**^*p*<0.05

The incidence of symptomatic AL was relatively low (2.7%) in this study, similar to two previous studies, which reported AL incidences of 2.2% and 3.6% [1, 6]. However, most studies reported the incidence of AL to be approximately 10–13% [3–5, 7]. This difference in AL rates is likely due to the inconsistent definition of AL, inclusion criteria of the studies, and different ratios of the patients who underwent rectal surgery. In fact, AL can be divided into symptomatic AL and asymptomatic AL, which does not require any treatment, and the standard classification criteria of AL (grades A, B, and C) advocated by ISREC are based on AL severity and the required treatment [15]. A high-quality prospective study by Qin [17] reported a total AL rate of 17% after LAR, with a clinical AL rate of 9.7%. In this study, nine (3.0%) asymptomatic patients who were evaluated as grade A were removed. This patient exclusion significantly reduced the overall incidence of AL. Moreover, the three colorectal surgeons in our unit have >20 years of surgical experience and have completely overcome the surgical learning curve, thus improving the safety of the operation. Furthermore, patients’ nutritional status was fully improved, and comorbidities such as diabetes and hypertension were effectively controlled before surgery. In the present study, all eight patients with symptomatic AL had no preoperative intestinal obstruction or elevated white blood cell count, and only one patient had hypoalbuminemia before surgery. This may also play an important role in preventing the occurrence of symptomatic AL in general.

With respect to the patient-related variables, the effects of neoadjuvant therapy on AL before surgery have been controversial in recent years. The effects of neoadjuvant therapy on AL before surgery have been controversial in recent years. Lim et al. [18] reported that neoadjuvant chemoradiation in lower rectal cancer was not associated with higher perioperative complications. By contrast, Park et al. [2] and Hamabe et al. [19] demonstrated that preoperative neoadjuvant chemotherapy with or without concomitant radiotherapy was associated with an elevated risk for AL with or without a defunctioning stoma after laparoscopic LAR. We believe that neoadjuvant therapy, particularly long-course radiotherapy, can cause tissue fibrosis, oligovascularization, tissue edema, and immunocompromise, which affect the healing of anastomoses and increase the incidence of postoperative symptomatic AL. Furthermore, a defunctioning stoma, an important protective factor, was often applied in patients who were given neoadjuvant therapy before surgery, which might minimize the consequence of symptomatic AL to a certain degree. In addition, all eight patients with symptomatic AL were younger (≤65 years) in the present study. This phenomenon was probably due to the small sample size of symptomatic AL, which was defined based on the proposal by ISREC. Therefore, we believe that if more patients were included in this study, the results might eventually change.

With respect to the surgery-related variables, the duration of operation was an independent risk factor associated with AL in previous studies [7, 20]. This study also demonstrated that prolonged operation time was associated with symptomatic AL. In a review of six patients who had prolonged operation times, we found that they were all males and 50% (3/6) of the patients received neoadjuvant therapy before surgery. The prolonged duration of operation may be caused by a relatively narrow pelvis in males, which leads to a more complicated operation than females. Although gender was not an independent risk factor, as determined by the multivariate analysis, the incidence of symptomatic AL in males was three times higher than the incidence of symptomatic AL in females (75% vs*.* 25%, *P* = 0.290). In addition, edema, the crispiness of the tissue, and the unclear anatomical level due to neoadjuvant therapy before surgery might increase the difficulty of surgery and might prolong the duration of operation. Therefore, it is necessary to pay attention to the possible causes of prolonged operation time. Numerous studies reported that the distance of the tumor to the anal verge was associated with AL [7, 19]. The present study revealed that the distance of the tumor to the anal verge was associated with symptomatic AL but was not an independent risk factor for symptomatic AL. The shorter distance of the tumor to the anal verge may result in poorer blood supply in the lower rectum and increased difficulties in performing surgery, both of which may increase the incidence of symptomatic AL.

The effects of two protective factors, namely, defunctioning stoma and transanal tube drainage, on the occurrence of AL are controversial. Of the 142 patients with defunctional stoma, two patients were evaluated as grade B and no defunctional stoma was performed in the nine patients diagnosed with grade A. Two previous studies revealed that an appropriate defunctioning stoma significantly decreases the occurrence of AL in high-risk patients [7, 19]. A recent high-quality meta-analysis involving 23 observational studies further concluded that no defunctioning stoma was a significant surgical related risk factor for AL [21]. However, Shiomi [22] and Salamone [8] reported that a defunctioning stoma did not decrease the occurrence of AL but did mitigate the clinical features and reoperation rate. In the present study, the symptomatic AL rate in the nondefunctioning stoma group (75%) was significantly higher than that in the defunctioning stoma group (25%), although the difference was not statistically significant (*P* = 0.193). This result may be due to the low incidence (2.7%) of symptomatic AL in the present study, which is a major limitation of the study. We were more preferred that the defunctioning stoma did not decreased the incidence of symptomatic AL, but it can reduce the risk of increasing the incidence of symptomatic AL due to early diarrhea after surgery. Moreover, the effect of a defunctioning stoma on quality of life, including the uncomfortable smell, demand for special care, and fecal dermatitis, should not be ignored. Therefore, to avoid unnecessary stoma, we believe that it is necessary to correctly identify high-risk patients of symptomatic AL before or during surgery. In addition, the criteria for placing transanal tube drainage have not been clearly defined. Tetsuo [23] reported that the transanal tube placement can prevent AL after laparoscopic LAR due to a reduction in the unfavorable incidence of early postoperative diarrhea. A recent meta-analysis also reported that patients in the transanal tube group tended to have lower reoperation rates and shorter hospital stays than patients in the nontransanal tube group [24]. In the present study, the symptomatic AL rate was higher in patients who underwent the transanal tube drainage than in those who did not, with no significant difference (87.5% vs. 12.5%, *P =* 0.994). However, this does not mean that the transanal tube drainage increased the probability of symptomatic AL in our unit. In contrast, this finding might imply that the three surgeons could more accurately predict the patients who were prone to symptomatic AL during in surgery.

With respect to a long-term survival, Kim et al. [10] reported that the 5-year overall survival rates according to each TNM classification did not differ between the leak and nonleak groups. Jang et al. [13] further revealed that AL was not associated with a significant increase in local tumor recurrence or a long-term survival after neoadjuvant therapy in rectal cancer. This study also did not find a relationship between symptomatic AL and overall survival. However, a recent meta-analysis with 35 studies reported that AL after anterior resection increased the risk of local recurrence and decreased the overall survival, cancer-specific survival, and disease-free survival [12]. A recent high-quality study including 900 patients also demonstrated that AL impairs the long-term survival in patients undergoing left-sided colorectal surgery [25]. Furthermore, long-term oncological outcomes are negatively affected by the occurrence of anastomotic leakage after rectal cancer surgery, which was demonstrated in the COLOR II study group [26]. This discrepancy might be due to the definition of AL, which has not been standardized. The definition of AL used in a study may have affected the outcome. Moreover, 37.5% (3/8) of patients with symptomatic AL in the present study received neoadjuvant therapy before surgery, which may have an impact on a long-term survival. Neoadjuvant therapy can decrease the tumor stage and improve local control after operation, which may affect the oncological outcomes more than the occurrence of AL [27, 28].

Two main limitations of this study should be considered. First, the incidence of symptomatic AL was much lower in this study than in most previous studies, which might hinder an adequate message from our research, such as age, diabetes, combined organ resection, and some tumor-related variables. Second, 58% of the included patients were ≤65 years old and 77% of them were evaluated as ASA II, which indicated the potential selection bias because of the retrospective nature of this study. Thus, more randomized controlled trials are needed in the future.

## Conclusion

In conclusion, the present study demonstrated that a prolonged duration of operation (≥200 min) is an independent risk factor for symptomatic AL after laparoscopic LAR for rectal cancer. An impact of symptomatic AL on 3- and 5-year overall survival was not observed in this study. Given the small number of patients who were identified as symptomatic AL, it is important to note that failure to detect a statistically significant difference between groups is not an adequate proof of equivalence. Further multicenter and randomized clinical trial studies are needed to explore patients with symptomatic AL in the future.

## Data Availability

The datasets used and/or analyzed during the current study are available from the corresponding author on reasonable request.

## Abbreviations

AL: Anastomotic leakage
LAR: Low anterior resection
ASA: American Society of Anesthesiologists
ISREC: International Study Group of Rectal Cancer
BMI: Body mass index
CEA: Carcinoembryonic antigen
χ^2^: Chi-squared test
CI: Confidence interval
